# Ozone Micro–Nano Bubbles Application Controls Disease Development and Maintains Quality of Fresh *Radix astragali*

**DOI:** 10.3390/jof12010044

**Published:** 2026-01-06

**Authors:** Yan Lv, Jihui Xi, Jinzhu Li, Cuixia Yang, Haijiao Chai, Huali Xue, Yang Bi

**Affiliations:** 1 College of Science, Gansu Agricultural University, Lanzhou 730070, China; 18435259652@163.com (Y.L.); lijz0708@163.com (J.L.); 18419087402@163.com (C.Y.); 15393386519@163.com (H.C.); 2 College of Food Science and Engineering, Gansu Agricultural University, Lanzhou 730070, China; xijihui2022@163.com (J.X.); biyang@gsau.edu.cn (Y.B.)

**Keywords:** *Radix astragali*, ozone micro–nano bubbles, postharvest diseases, active ingredients, metabolomic analysis

## Abstract

Ozone micro–nano bubbles (OMNBs) are an emerging preservation technology. However, there are few reports regarding their application in controlling postharvest diseases of agricultural products. *Radix astragali*, as a medicinal and edible plant, is particularly vulnerable to pathogenic microorganisms during postharvest storage, which leads to diminishing the quality and commercial value. In this study, fresh *R. astragali* inoculated with *Penicillium polonicum* was treated with different concentrations (2, 3, 4, 5, 6, 8 mg/L) of OMNBs and stored at room temperature for 28 days. The results indicate that 3 mg/L OMNBs application for 8 min effectively inhibited the development of blue mold in fresh *R. astragali* and preserved its quality. Then, we compared the three different treatments of micro–nano bubbles (MNBs), 3 mg/L O_3_, and 3 mg/L OMNBs on physiological and pathological parameters of un-inoculated fresh *R. astragali* during storage and analyzed the changes in the active ingredients by liquid chromatography and metabolomics. The results indicate that the 3 mg/L OMNBs treatment effectively inhibited the decline in weight loss rate, respiratory rate, firmness, browning index, and ABTS and DPPH radical-scavenging rates, as well as reduced the incidence rate and disease index of fresh *R. astragali* during storage. The metabolomics results suggest that the 3 mg/L OMNBs application activated the mevalonate pathway (MVA), the methylerythritol phosphate pathway (MEP), and the phenylpropanoid biosynthesis pathway to maintain the content of active ingredients such as terpenoids and flavonoids, and these findings are consistent with the results of HPLC-MS analysis.

## 1. Introduction

*Radix astragali* (*R. astragali*), commonly known as yellow astragalus or northern astragalus, belongs to the legume family, which is widely used as a qi-tonifying herb in traditional Chinese medicine and regarded as the “chief of qi tonics”. *R. astragali* has pharmacological functions of tonifying qi and strengthening the exterior, promoting diuresis and reducing edema, detoxifying, and facilitating tissue repair due to its properties that include a warm nature and sweet taste, where it enters the spleen and lung meridians [1]. Modern pharmacological studies indicate that *R. astragali* contains various bioactive compounds, including astragalus polysaccharides, saponins, and flavonoids [2], which result in anti-inflammatory, antioxidant, antitumor, and cardio-protective activities [3]. Since *R. astragali* was listed as a medicinal and edible plant, its cultivation area has increased at an unprecedented scale. *R. astragali* is traditionally processed into drying medicinal slices; however, there is a significant loss in active components during the drying process. Studies have demonstrated that fresh *R. astragali* can better avoid the loss of the functional bioactive components compared to traditional dried slices [4].

Nevertheless, fresh *R. astragali* is prone to decay during postharvest storage due to pathogenic organisms such as root rot caused by *Fusarium solani* [5] and gray mold caused by *Botrytis cinerea* [6]. Our group previously identified *Penicillium polonicum* as the predominant pathogen responsible for blue mold in fresh *R. astragali* during postharvest storage: when cultured on a PDA medium, isolates of *P. polonicum* obtained from infected astragalus tissues initially appeared white, gradually deepening to a dark olive-green over time, exhibiting typical morphological characteristics of the Penicillium genus [7]. Studies have revealed that *P. polonicum* is widely distributed in grains, peanuts, dried meat, and citrus fruits and is an important producer of mycotoxins [8]. This not only leads to a decline in product quality and commercial value but also poses health risks due to potential mycotoxin contamination.

To effectively control postharvest decay of fruits and vegetables caused by pathogens such as *P. polonicum*, researchers have developed various control strategies. In terms of biological control, yeast and its derivatives show potential. For example, treatment with yeast cell wall polysaccharides can reduce the incidence of blue mold caused by Penicillium expansum in peach fruits and induce an increased activity of defense-related enzymes as well as a higher total phenolic content [9,10]. Plant-derived extracts also exhibit in vitro antifungal activity; for instance, extracts from Larrea tridentata inhibit *P. polonicum* [11], and phenolic extracts from myrtle leaves show bactericidal effects against *P. digitatum*, *P. italicum*, and *P. expansum* [12]. Additionally, cinnamaldehyde treatment can suppress infection of citrus by *P. digitatum* and *Geotrichum citri-auranti* through systemically induced resistance [13]. Physical control methods such as negative air ion (NAI) technology can inhibit *Penicillium citrinum*, reduce decay rates in bayberry fruits, and help to maintain fruit quality [14]. However, these biological and physical approaches often face limitations, including high costs for large-scale production, insufficient stability, and limited environmental adaptability. Currently, chemical fungicides are the primary strategy for controlling blue mold in recent decades; nevertheless, there is a rise in issues concerning the emergence of resistant strains coupled with chemical residues and environmental pollution due to the excessive application of the chemicals. Therefore, the need for a safe, eco-friendly, sustainable preservation strategy to combat blue mold is urgently intensified.

Ozone, a potent oxidizing gas, exhibits significant potential for applications in food preservation. It achieves effective microbial inactivation by disrupting microbial cell membrane structures and oxidizing key metabolic enzymes, while also suppressing physiological processes such as ethylene biosynthesis, thereby delaying postharvest senescence in fruits and vegetables [15,16]. However, limitations of conventional ozone treatments—including low solubility, uneven distribution, and the risk of oxidative damage at elevated concentrations [17]—constrain its safe and efficient application in certain food systems.

Micro–nano bubbles (MNBs) technology offers a promising approach for enhancing the utility of ozone in food preservation, owing to its high specific surface area, prolonged aqueous-phase retention, and distinctive gas–liquid interfacial properties [18]. By dispersing ozone into microbubbles and nanobubbles, this technology markedly increases the gas–liquid contact area and extends the residence time of ozone in aqueous media [19], thereby improving antimicrobial and preservative efficacy even at lower ozone concentrations.

In recent years, ozone micro–nano bubbles (OMNBs) have demonstrated notable advantages in food preservation. Compared with conventional ozone treatments, OMNBs not only enhance ozone mass transfer and distribution homogeneity but also reduce oxidative stress risks. Numerous studies suggested that 2.5 mg/L OMNBs can effectively alleviate the senescence and yellowing of parsley during storage [20]. Hou et al. [21] suggested that OMNBs application not only reduced the number of *Escherichia coli* in Napa cabbage but also efficiently degraded the pesticide of acetamiprid. However, the application of OMNBs in the preservation of medicinal and edible plants remains underexplored, particularly regarding their mechanisms for protecting postharvest quality and preserving the bioactive constituents of herbal materials such as *R. astragalus.*

To address this knowledge gap, we advance the hypothesis that OMNBs, at a specific concentration, can systematically regulate the physiological metabolism of *R. astragalus*, thereby inhibiting pathogen growth while maintaining host tissue health. To test this hypothesis, this study aimed to systematically investigate the effects of OMNBs treatment on the physiological metabolism and disease progression of postharvest *R. astragalus* using an integrated multi-omics strategy. We first screened the optimal concentration of OMNBs and evaluated their efficacy in controlling blue mold caused by *P. polonicum* in fresh *R. astragali*. Subsequently, the effects of O_3_, MNBs, and OMNBs treatments on the postharvest physiology, pathology, quality, and bioactive components of fresh *R. astragali* were compared. Metabolomic analysis was further employed to elucidate the mechanism by which OMNBs treatment preserves the active constituents of *R. astragali*. This study is expected to provide a theoretical basis for the application of OMNBs technology in the postharvest preservation of medicinal plants and to offer new insights for developing efficient and environmentally friendly storage technologies for Chinese herbal medicines.

## 2. Materials and Methods

### 2.1. Preparation of OMNBs

Micro–nano bubbles (MNBs) were generated using a micro–nano bubble generator (RJI-MIN-300, Shanghai, China). The water inlet flow rate was adjusted to 8 L min^−1^ and a milky white, emulsion-like MNBs suspension was obtained. Ozone (O_3_) was produced by using an oxygen-source ozone generator (RJC-YC-20, Shanghai, China). The generator was operated with an air inlet flow of 6 L min^−1^, which then yielded an ozone output of 8 mg h^−1^. The ozone gas was subsequently introduced into the micro–nano bubbles generator to produce ozone micro–nano bubbles (OMNBs) at a concentration of 8 mg L^−1^.

**Figure 1:**
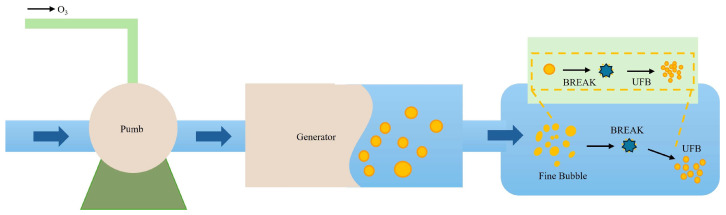
The working principle of the OMNBs system. Pump: Delivers liquid into the system. Generator: Device responsible for generating ultrafine bubbles (UFBs). UFBs: Ultrafine bubbles, also referred to as micro–nano bubbles, produced by the system. Fine Bubble: Synonymous with UFBs, indicating nanoscale gaseous dispersions in the liquid.

The working principle of the OMNBs system is illustrated in Figure 1. Ozone and water entered the generator through a water pump, where a dynamic high-speed shearing unit dispersed the gas into micro- and nanometer-sized bubbles. The intense shear forces fragmented the gas phase to diameters of 10–100 nm, ultimately forming a stable, milky white suspension of ozone micro–nano bubbles.

### 2.2. Screening of Optimal Treatment Concentration of OMNBs

Fresh *Radix astragali* samples were collected from the *R. astragali* cultivation base in Min County, Dingxi City, Gansu Province, China on November 2024. Roots with uniform size and thickness, free from mechanical damage or decay, were selected and transported to the Chemical Biology Laboratory of the College of Science, Gansu Agricultural University within 8 h after harvest. The roots were cut into 8 cm segments, surface-sterilized in 1% (*v*/*v*) sodium hypochlorite (NaClO) solution for 5 min, and rinsed thoroughly with sterile distilled water to remove residual NaClO. The samples were then air-dried under sterile conditions.

*Penicillium polonicum* was isolated from the blue mold of *R. astragali* and characterized by morphological and molecular biological techniques by our research group, and then the spore suspension (1 × 10^6^ CFU mL^−1^) of *P. polonicum* was evenly sprayed onto the surface of the *R. astragali* samples. After 10 h of inoculation, the inoculated *R. astragali* samples were immersed in ozone micro–nano bubbles (OMNBs) solutions at concentrations of 2, 3, 4, 5, 6, and 8 mg L^−1^ for 8 min. The treated samples were subsequently air-dried, then sealed in sterile plastic containers, and then stored at room temperature (25 ± 1 °C) for 28 days.

#### 2.2.1. Weight Loss Rate

In the process of storage, *R. astragali* was weighed regularly by electronic balance (AL-104, Shanghai, China).

$$\mathrm{Weight}\;\mathrm{loss}\;\mathrm{rate}\;(\%)=\frac{\mathrm{initial}\;\mathrm{mass}-\mathrm{detection}\;\mathrm{mass}\;\mathrm{of}\;\mathrm{the}\;\mathrm{day}}{\mathrm{initial}\;\mathrm{mass}}\;\times \;100$$

#### 2.2.2. Browning Index

The color change of *R. astragali* was measured by a colorimeter (CS-210, Hangzhou, China), and the browning index (BI) of *R. astragali* was calculated according to the recorded L*, a*, and b* values. The calculation formula is as follows [22]:

Browning index (BI) = (x − 0.31) × 100/0.172

x=(a*+1.75 × L*)/(5.645 × L*+a* − 3.012 × b*)

where L*, a*, and b* represent the lightness, red–green, and yellow–blue coordinates of the color space, respectively.

#### 2.2.3. Incidence Rate

During the whole storage, the surface color of *R. astragali* manifested dark black or brown spots, with soft rotten, wrinkled, and mildew symptoms, whose phenomena are regarded as due to the incidence of *R. astragali* [23].

$$\mathrm{Incidence}\;\mathrm{rate}\;(\%)=\frac{\mathrm{diseased}\;\mathrm{plant}}{\mathrm{observed}\;\mathrm{total}\;\mathrm{plant}}\;\times \;100$$

### 2.3. Effects of Different OMNBs Treatments on the Physiology, Pathology, and Quality of Fresh R. astragali

#### 2.3.1. Sample Pretreatment

Fresh *R. astragali* roots without visible mechanical damage and with uniform thickness were selected and cut into 8 cm segments. The samples were surface-sterilized in 1% (*v*/*v*) sodium hypochlorite (NaClO) solution for 5 min, rinsed thoroughly with sterile distilled water to remove residual NaClO, and air-dried under sterile conditions.

The sterilized roots were then divided into four treatment groups: (i) fumigation with 3 mg L^−1^ ozone (O_3_) for 0.5 h; (ii) immersion in 3 mg L^−1^ ozone micro–nano bubbles (OMNBs) for 8 min; (iii) immersion in 3 mg L^−1^ micro–nano bubbles (MNBs) for 8 min; and (iv) treatment with distilled water as the control. After treatment, the samples were naturally air-dried and sealed in transparent plastic boxes at room temperature. Subsamples were collected at 0, 7, 14, 28, 42, and 56 days for photographic documentation and analysis of physiological, pathological, and quality parameters. For the experiment, three diseased roots were randomly selected from each treatment group (three biological replicates; nine roots in total) and then ground into fine powder in the presence of liquid-nitrogen and stored at −80 °C for subsequent analyses.

#### 2.3.2. Physiology and Quality Assay

##### Weight Loss Rate and Browning Index

The weight loss rate and the browning index were separately determined based on the abovementioned methods of Section 2.2.1 and Section 2.2.2.

##### Respiratory Rate and Firmness

The respiration rate of *R. astragali* was measured based on the static method [24]. Eight *R. astragali* roots with uniform size were placed in airtight glass jar with a volume of 2 L for 20 min. The concentration of CO_2_ was measured by a portable CO_2_ respiratory detector. The calculation formula of respiration intensity is as follows:

$$\mathrm{respiration}\;\mathrm{rate}\;(\mathrm{mg}\;{\mathrm{CO}}_{2}\;/(\mathrm{kg}*h)\;=\frac{(N_{2}\;-\;N_{1})\;\times \;1.796\;\times \;V}{m\;\times \;t}\;\times \;100$$

where N_1_ and N_2_ represent the concentration of CO_2_ before and after the detection the unit of N_1_ and N_2_ (ppm), V represents the volume of the airtight container (L), m represents the mass of *R. astragali* mongholicus (g), and t represents the determination time (h).

The firmness of *R. astragali* was measured by using a fruit hardness tester (GY-4, Wenzhou, China). The maximum force was expressed as N, and each treatment included three replicates.

##### Determination of Antioxidant Activity

The ABTS radical-scavenging activity was determined according to the method of Chen et al. [25] with minor modifications. Briefly, 7 mM ABTS and 2.45 mM K_2_S_2_O_8_ solutions were mixed at a 1:1 ratio and incubated in the dark for 12–16 h to generate ABTS^+^• radical cations. The obtained solution was diluted with phosphate-buffered saline (PBS) until an absorbance of 0.70 ± 0.02 at 734 nm was obtained, which was denoted as A_0_. Then, 20 μL of 1 mg mL^−1^ *R. astragali* extract was added to 980 μL ABTS working solution and allowed to react for 6 min at room temperature in the dark. The absorbance of the reaction mixture was measured at 734 nm using a spectrophotometer (UV-1800, Shimadzu Corp., Tokyo, Japan) and recorded as A_X_.

$$\mathrm{ABTS}\;\mathrm{radical-scavenging}\;\mathrm{rate}\;\left(\%\right)=\left(1\;-\;\frac{A_{X}}{A_{0}}\right)\times 100$$

The DPPH radical-scavenging activity was determined following the method of Ranggainin et al. [26] with slight modifications. DPPH (20 mg) was dissolved in anhydrous ethanol and made up to 250 mL to obtain a 0.1 mM DPPH working solution, which was then stored at 4 °C in the dark until use. Then, 0.1 mg mL^−1^ *R. astragali* extract (0.1 mL) was mixed with 0.9 mL of the DPPH solution and incubated in the dark for 30 min. The absorbance was measured at 517 nm (Aᵢ). The absorbance of a blank solution containing 1 mL DPPH and 5 mL ethanol was measured as Aⱼ.

$$\mathrm{DPPH}\;\mathrm{radical-scavenging}\;\mathrm{rate}\;\left(\%\right)=\left(1\;-\;\frac{A_{i}}{A_{j}}\right)\times 100$$

#### 2.3.3. Incidence Rate and Disease Index

The incidence rate was determined based on the abovementioned method of Section 2.2.3.

The disease index statistics was determined by Huang et al. [27] with some modifications, and the incidence of *R. astragali* was graded (Table A1) to calculate the disease index (DI) of *R. astragali*:

$$\mathrm{Disease}\;\mathrm{index}\;(\mathrm{DI})\;=\;\frac{\sum \mathrm{Plants}\;\mathrm{with}\;\mathrm{each}\;\mathrm{disease}\;\times \;\mathrm{disease}\;\mathrm{grade}}{\mathrm{Total}\;\mathrm{plants}\;\times \;\mathrm{highest}\;\mathrm{disease}\;\mathrm{grade}}\;\times \;100$$

#### 2.3.4. Active Ingredients Analysis of *R. astragali*

In the experiment, the treated *R. astragali* samples stored for 7, 14, 28, 42, and 56 days were employed for the determination of active components. The ten bioactive compounds comprise astragaloside I, astragaloside II, astragaloside III, astragaloside IV, calycosin, calycosin-7-glucoside, formononetin, ononin, 7,2′-dihydroxy-3′,4’-dimethoxyisoflavan, and 3-hydroxy-9,10-dimethoxypterocarpan (Table A2), which were determined and quantified by using an ultra-performance liquid chromatography–mass spectrometry (UPLC-MS) system (Agilent 1290-6460, Santa Clara, CA, USA) following the method of Xi et al. [28] with minor modifications.

Approximately 1.0 g of each treated *R. astragali* sample was accurately weighed and placed into a conical flask. A total of 25 mL methanol was added, and the mixture was extracted by ultrasonic treatment for 30 min (100 W, 40 kHz, 35 °C). The extract was filtered, and 5 mL of the filtrate was transferred into a 10 mL volumetric flask. Then, 100 μL internal standard solution was added, and the volume was made up to the mark with methanol. The solution was thoroughly mixed, filtered through a 0.22 μm organic microporous membrane, and determined by UPLC–MS. Each sample was measured in triplicate in the experiment.

#### 2.3.5. Metabolomics Analysis

The treated *R. astragali* samples from the control group (day 0), the sterilized water (day 42), MNBs (day 42), O_3_ (day 42), and OMNBs (day 42) were collected for metabolomic analysis. A total of five groups of samples were analyzed, each in triplicate. Metabolomic profiling was performed using liquid chromatography–tandem mass spectrometry (LC–MS/MS). Data processing and statistical analysis were conducted in the R environment using the MetaboAnalystR package (version 2.0.1) [29].

Raw data were subjected to quality-control (QC) evaluation, batch correction, and normalization prior to statistical analysis. Basic statistical evaluation and visualization of metabolite abundance were carried out using hierarchical clustering heatmaps, histograms, and principal component analysis (PCA). To identify differential metabolites, partial least squares discriminant analysis (PLS-DA), orthogonal PLS-DA (OPLS-DA), volcano plot analysis, and machine-learning-based feature selection methods were applied.

Functional interpretation was further performed. Pathway analysis integrated enrichment and topological analyses to determine key metabolic pathways involved in the biological responses under study.

### 2.4. Statistical Analysis

The study followed a completely randomized design with three biological replicates per treatment. Each biological replicate was defined as an independent container stocked with Astragalus roots. At each sampling time point, three roots were randomly selected from each container, yielding nine independent root samples per treatment (3 containers × 3 roots). Since all physiological parameters were measured on individual roots, the statistical sample size was *n* = 9 per treatment. Data are expressed as the mean ± standard deviation (SD). Differences among multiple groups were analyzed by one-way analysis of variance (one-way ANOVA) followed by Fisher’s least significant difference (LSD) post hoc test for pairwise comparisons using IBM SPSS Statistics software (version 27.0; IBM Corp., Armonk, NY, USA). Differences between treatments were considered statistically significant at *p* < 0.05, *p* < 0.01, and *p* < 0.001; “ns” indicates no significant difference. Figures were generated using Origin software (version 2024; OriginLab Corp, Northampton, MA, USA).

## 3. Results and Analysis

### 3.1. Screening of Optimal Concentration of OMNBs

**Figure 2:**
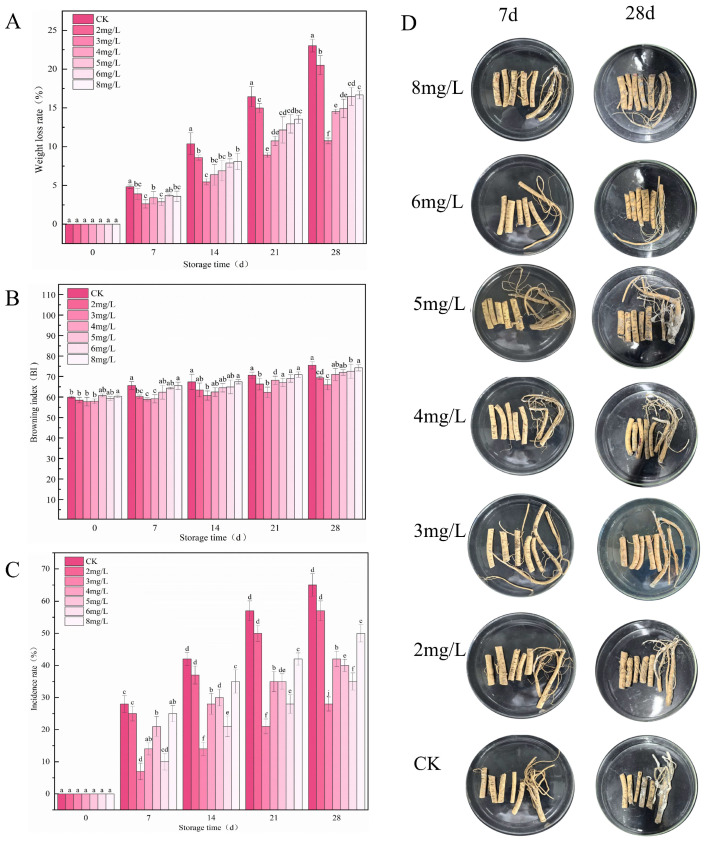
Effects of different concentrations of OMNBs on weight loss rate (**A**), browning index (**B**), incidence rate (**C**), and appearance quality (**D**) in *R. astragali* inoculated with *Penicillium polonicum* during storage. Letters a, b, c, d, e, f, j above the columns are significance markers. Identical letters indicate no significant difference between groups, while different letters indicate significant differences. Multiple letters (e.g., “ab”) indicate that the group has no significant difference with any group containing either of those letters.

Analysis of fresh *R. astragali* tissues inoculated with *P. polonicum* and treated with different concentrations of ozone micro–nano bubbles (OMNBs) revealed that the weight loss rate, browning index, and incidence rate were gradually enhanced as the storage time increased (Figure 2A–C). However, 3 mg L^−1^ OMNBs markedly inhibited blue mold of *R. astragali* extension (Figure 2D), and the increases in weight loss rate, browning index, and incidence rate were significantly lower than those in other treatments. For instance, the weight loss rate and incidence rate of the samples treated with 3 mg L^−1^ OMNBs were 10.75% and 54.17%, respectively. Nevertheless, 2, 4, 5, 6, and 8 mg L^−1^ OMNBs application resulted in 20.51%, 14.53%, 14.93%, 16.48%, and 16.67% for weight loss and 75.00%, 54.17%, 70.83%, 79.17%, and 66.67% for incidence rate, respectively, on day 28. In addition, the browning index of samples treated with 3 mg L^−1^ OMNBs increased only slightly, with a change from 57.73 to 65.97, whereas those of other treatments increased by 20.62%, 15.72%, 18.40%, 15.63%, and 18.38%, respectively (Figure 2), indicating that 3 mg L^−1^ OMNBs effectively suppressed *P. polonicum* infection and delayed the quality deterioration of *R. astragali* during postharvest storage.

### 3.2. Effects of Different Treatments on Physiology and Quality of Fresh R. astragali

**Figure 3:**
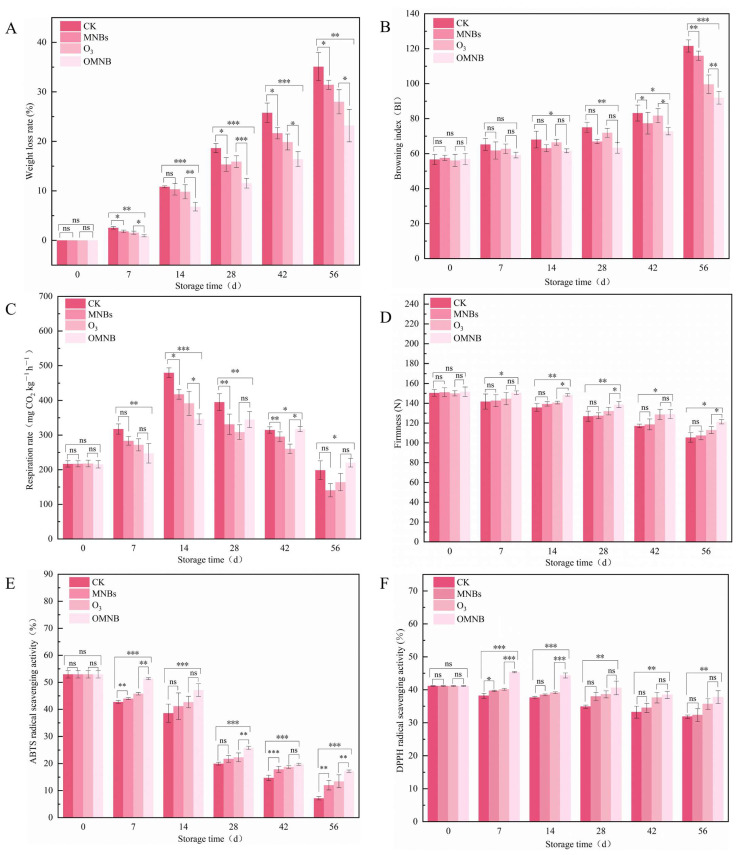
Effects of different treatment groups on weight loss rate (**A**), browning index (**B**), respiratory rate (**C**), firmness (**D**), ABTS (**E**), and DPPH (**F**) in non-inoculated *R. astragali* during storage. The meaning of the significance markers in the figure is as follows: * *p* < 0.05, ** *p* < 0.01, *** *p* < 0.001, ns (not significant, *p* ≥ 0.05).

The weight loss rate increased gradually during storage for all treated *R. astragali* (Figure 3A). On day 56, the weight loss rate in the control group reached 35.09%, whereas samples treated with MNBs, O_3_, and OMNBs exhibited lower values of 31.42%, 27.98%, and 23.19%, respectively, among which, OMNBs treatment significantly suppressed weight loss throughout the storage period.

The browning index also manifested a continuous upward trend (Figure 3B). However, the OMNBs-treated samples consistently exhibited the lowest browning change among all treatments. Severe browning was observed in the control and MNBs-treated groups at the later stages of storage, with browning indices of 121.55 and 115.98 on day 56, respectively. In contrast, the browning index of the OMNBs treatment was only 91.93. Notably, the O_3_-treated samples showed greater browning than the MNBs-treated samples during early storage, especially on day 28, with browning indices of 71.89 and 66.90, respectively.

The respiration rate of *R. astragali* firstly increased and then declined during the whole storage (Figure 3C). The OMNBs-treated samples maintained a relatively low and stable respiration rate, reaching a peak of 346.01 mg CO_2_ kg^−1^ h^−1^ on day 14. The respiration rates of the control, MNBs, and O_3_ treatments were 178.55, 200.88, and 183.24 mg CO_2_ kg^−1^ h^−1^, respectively, while the OMNBs-treated samples exhibited the lowest value of 166.03 mg CO_2_ kg^−1^ h^−1^ on day 56.

Tissue firmness decreased gradually in all groups during the whole storage (Figure 3D). From day 0 to 56, firmness in the control group decreased by 29.90%. Under the same storage conditions, samples treated with O_3_ and MNBs showed reductions of 24.78% and 28.87%, respectively, whereas OMNBs treatment resulted in the smallest decline of 19.99%.

Both ABTS and DPPH radical-scavenging activities decreased throughout the storage period (Figure 3E,F). On day 56, ABTS radical-scavenging activities in the control, MNBs, and O_3_ treatments were 7.16%, 11.99%, and 13.42%, respectively, while the corresponding DPPH activities were 31.82%, 32.30%, and 35.66%. In comparison, OMNBs-treated samples retained higher antioxidant capacities, with ABTS activity of 17.19% (2.1 times that of the control) and DPPH activity of 37.92%, both exceeding those of the O_3_ and MNBs treatments.

These findings demonstrate that OMNBs treatment effectively delayed weight loss, browning, and tissue softening, stabilized respiration, and preserved antioxidant activity in fresh *R. astragali* during postharvest storage.

### 3.3. Effects of Different Treatments on Disease Control of Fresh R. astragali

The mildew process of *R. astragali* during storage could be divided into three distinct stages. During the first stage (0–7 days), reddish-brown spots appeared on the surfaces of samples in the control, MNBs, and O_3_ treatments, but no visible decay or mold growth was observed. The second stage (7–42 days) was characterized by the gradual appearance of white mycelia on all samples. Mold development was most severe in the control group, which displayed clear signs of decay and darkening. Moderate infection occurred in the O_3_ and MNBs treatments, although mold growth appeared earlier in the MNBs group than in the O_3_ group. In contrast, only a small amount of superficial white mold was observed in the OMNBs-treated samples, and no evident decay was observed. In the third stage (42–56 days), softening and extensive rotting were observed in the control and MNBs groups. White mold continued to increase in the O_3_ and OMNBs treatments, but the degree of mildew and tissue decay in the OMNBs group remained substantially lower than in all other treatments.

**Figure 4:**
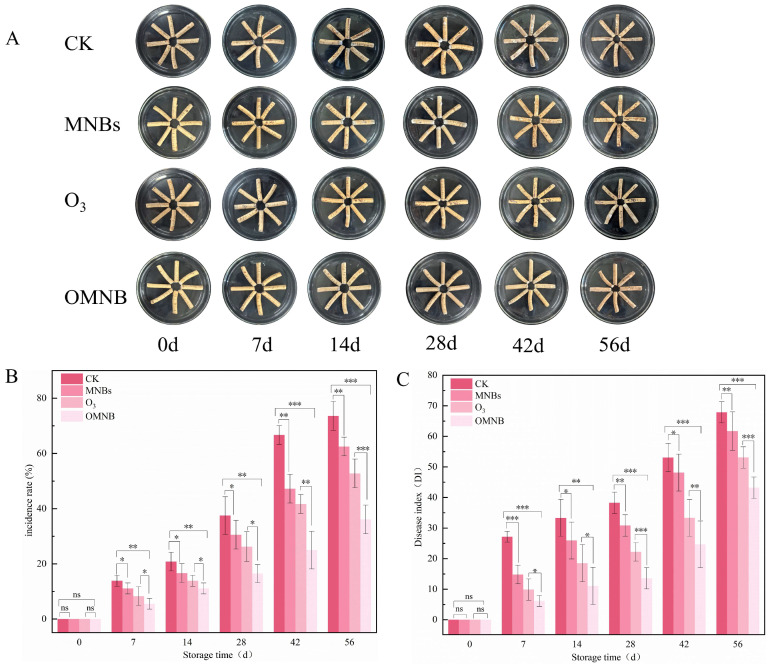
Effects of different treatment groups on appearance quality (**A**), incidence rate (**B**), and disease index (**C**) in non-inoculated *R. astragali* during storage. The meaning of the significance markers in the figure is as follows: * *p* < 0.05, ** *p* < 0.01, *** *p* < 0.001, ns (not significant, *p* ≥ 0.05).

The disease incidence of *R. astragali* increased in all groups throughout the storage (Figure 4A). On day 56, the disease incidence rate in the control group reached 73.16%, which was 2.04 times higher than that of the OMNBs treatment. The incidence rates of the O_3_ and MNBs treatments increased to 62.50% and 52.77%, respectively, both significantly greater than that in the OMNBs group.

The disease index was consistent with incidence rate, which also increased gradually during the whole storage (Figure 4B). On day 56, the disease index of the OMNBs-treated samples remained the lowest (43.21), followed by the O_3_ (53.09), MNBs (61.71), and control (67.86) groups, which indicate that OMNBs treatment effectively inhibited fungal growth and delayed tissue decay of *R. astragali* during postharvest storage.

### 3.4. Effect of Different Treatments on the Active Ingredients of Fresh R. astragali

UPLC–MS was employed to analyze the change in the ten major active compounds in fresh *R. Astragali* after different treatments (Table A2). Among these, the contents of astragaloside I, astragaloside II, astragaloside III, astragaloside IV, calycosin, calycosin-7-glucoside, formononetin, 7,2′-dihydroxy-3′,4′-dimethoxyisoflavan, and 3-hydroxy-9,10-dimethoxypterocarpan initially increased and then declined during storage; most of these compounds, including astragaloside I, calycosin, calycosin-7-glucoside, and formononetin, attained their maximum concentrations on day 42. In contrast, the contents of astragaloside II and astragaloside III displayed an overall decreasing trend, whereas astragaloside IV and formononetin exhibited gradual increases throughout the storage.

For the OMNBs-treated samples, the contents of astragaloside I, astragaloside IV, formononetin, calycosin-7-glucoside, and 3-hydroxy-9,10-dimethoxypterocarpan were higher than those in the other groups, reaching 1676.36 µg g^−1^, 128.63 µg g^−1^, 114.55 µg g^−1^, 86.42 µg g^−1^, and 57.45 µg g^−1^, respectively, on day 42. However, the contents of astragaloside II and astragaloside III in the OMNBs treatment were relatively lower, with 11.99 µg g^−1^, 17.57 µg g^−1^, and 14.68 µg g^−1^, respectively, compared with those in the other treatments.

According to the Chinese Pharmacopoeia, astragaloside IV and calycosin-7-glucoside are established as the pivotal marker compounds for quality control of the *R. astragalus* herb and its preparations [30]. Astragaloside IV, the most extensively studied triterpenoid saponin, exhibits a wide range of pharmacological activities, including immunomodulation, anti-fatigue, anti-tumor, and cardio-protective as well as reno-protective effects [31,32]. Concurrently, calycosin-7-glucoside, a key flavonoid, demonstrates potent antioxidant, anti-inflammatory, cardiovascular-protective, and neuro-protective effects [33,34]. Thus, among the five components with elevated concentrations in the OMNBs group, astragaloside IV and calycosin-7-glucoside hold primary importance in terms of pharmacopoeial functions.

These results indicate that OMNBs treatment effectively maintained or promoted the accumulation of key bioactive components in *R. astragali*, particularly major triterpenoid saponins and isoflavonoids, thereby contributing to improved postharvest quality and pharmacological value.

### 3.5. Effects of Different Treatments on Metabolite Content

**Figure 5:**
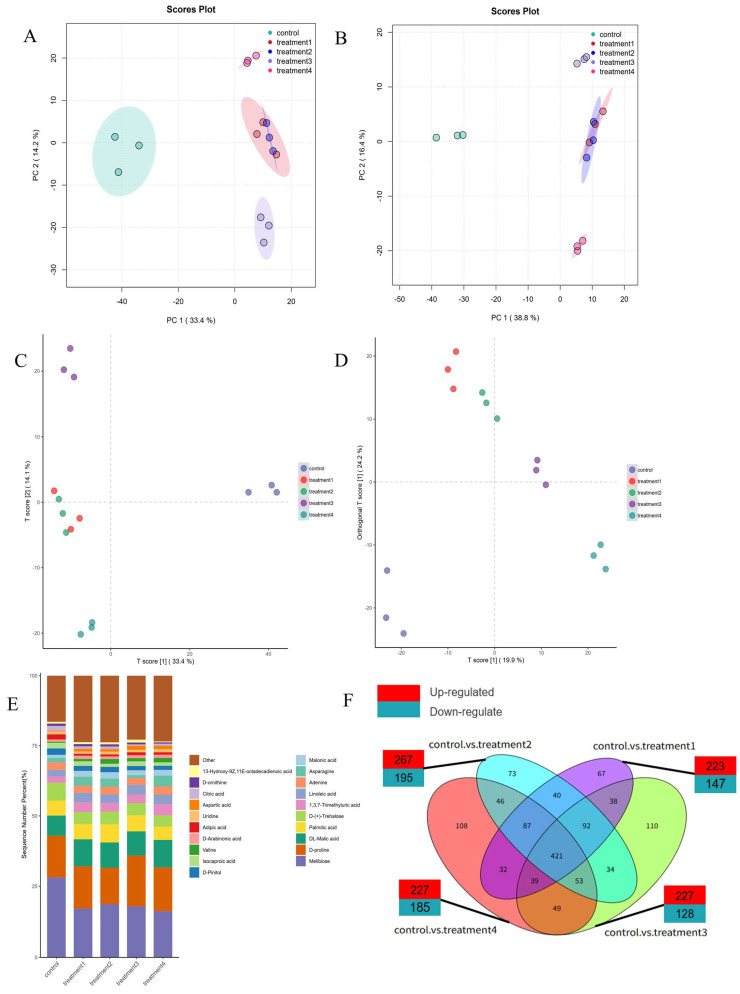
PCA diagram in positive ion mode (**A**), PCA diagram in negative ion mode (**B**), PLS-DA diagram (**C**), and DPLS-DA diagram (**D**); the column chart of the percentage accumulation of the top 20 metabolites in different treatment groups (**E**). The difference metabolites Venn diagram of *R. astragalus* on day 0 and CK group, MNBs, O_3_, and OMNBs treatments on the 42nd day. (**F**) Control represented the sample on day 0, treatment 1 represented the control group on the 42nd day (42d-CK), treatment 2 represented the MNBs treatment on the 42nd day (42d-MNBs), treatment 3 represented the O3 treatment on the 42nd day (42d-O_3_), and treatment 4 represented the OMNBs treatment on the 42nd day (42d-OMNBs).

Principal component analysis (PCA) was performed to evaluate the overall metabolic variation among treatments and the degree of consistency within groups [35]. The first two principal components (PC1 and PC2) explained 47.6% of the total variance in both positive (Figure 5A) and negative (Figure 5B) ionization modes. The score plots clearly separated the different treatment groups, indicating distinct metabolic profiles. Moreover, samples within each treatment clustered closely together, demonstrating good analytical stability and reproducibility. A slight overlap was observed between treatment 1 and treatment 2, suggesting similar metabolic characteristics between these two groups. Partial least-squares discriminant analysis (PLS-DA; Figure 5C) and orthogonal PLS-DA (OPLS-DA; Figure 5D) further confirmed the clustering patterns, validating the reliability of the metabolomic data for subsequent analysis.

A total of 2068 differential metabolites (DMs) were identified across the combined positive and negative ionization modes. The bar chart illustrates the top 20 most abundant metabolites, highlighting clear compositional differences among treatments. The predominant metabolites detected in *R. astragali* included DL-arginine, choline cation, arginine, and so on (Figure 5E). Notably, the metabolite profile of the samples subjected to OMNBs treatment for 42 days closely resembled the freshly harvested (day 0) *R. astragali*. The levels of the metabolites, detailed in Table A3, reveal that DL-arginine, choline cation, arginine, melibiose, and D-proline account for 10.08%, 9.08%, 6.25%, 7.24%, and 3.97% in the control group, versus 10.90%, 8.49%, 5.60%, 5.49%, and 3.96% in the treatment 4 group. This closer resemblance to baseline levels (day 0) suggests a superior efficacy of OMNBs in conserving the compositional integrity of *R. astragali*.

### 3.6. Effects of Different Treatments on Metabolic Pathways Related to Active Ingredients

The metabolomic profiles of the 42-day treatments (CK, MNBs, O_3_, and OMNBs) were separately compared with those of the untreated samples at day 0. As shown in Figure 5F, 379 differential metabolites (DMs) were identified in the 42d-CK vs. 0d comparison (232 up-regulated and 147 down-regulated), 462 DMs were identified in 42d-MNBs vs. 0d (267 up-regulated and 195 down-regulated), 355 DMs were identified in 42d-O_3_ vs. 0d (227 up-regulated and 128 down-regulated), and 412 DMs were identified in 42d-OMNBs vs. 0d (227 up-regulated and 185 down-regulated).

Among these DMs, astragaloside I and astragaloside IV were significantly up-regulated in the 42d-OMNBs groups, whereas astragaloside II and astragaloside III were markedly down-regulated in 42d-OMNBs (Table 1). These findings were consistent with the UPLC-MS results of active ingredient analysis (Table A2), confirming that OMNBs treatment maintained higher levels of key saponins such as astragaloside I and astragaloside IV.

**Figure 6:**
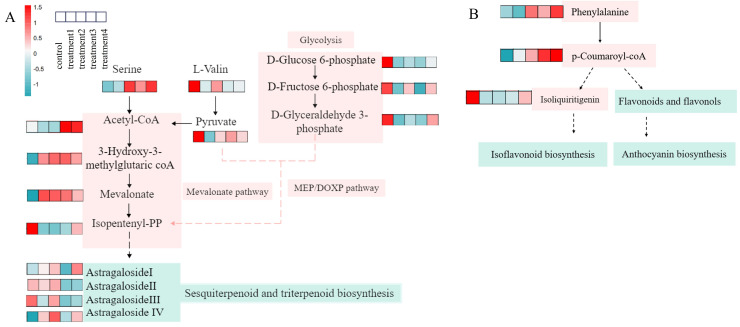
Terpenoid biosynthesis pathway diagram and related DM regulation heat map (**A**); flavonoid biosynthesis pathway and related DM regulatory heat map (**B**). The solid arrows in the figure represent direct or major metabolic transformation steps; the dashed arrows indicate multi-step, regulatory, or summarized auxiliary pathways.

Astragaloside I, II, III, and IV are triterpenoid saponins derived from the terpenoid metabolic pathway. KEGG enrichment analysis of these four differential metabolites (VIP > 1, *p* < 0.05) indicated that isopentenyl pyrophosphate (IPP)—a key precursor for terpenoid biosynthesis—can be produced via both the mevalonate (MVA) and methylerythritol phosphate (MEP) pathways (Figure 6A). In the MVA pathway, acetyl-CoA serves as the initial substrate, whereas the MEP pathway is initiated from glycerol 3-phosphate and pyruvate. In addition, D-glucose 6-phosphate can be converted to glycerol 3-phosphate through glycolysis, and amino acids such as DL-serine and DL-valine can be catabolized to acetyl-CoA and pyruvate, thereby influencing the biosynthesis of triterpenoid saponins.

Saponins are the principal bioactive components of *R. astragali* and are known for their antioxidant and anti-inflammatory activities. The significant upregulation of astragalosides I and IV in the OMNBs treatment suggests that OMNBs effectively maintained the biosynthetic activity of these functional compounds, thereby enhancing the medicinal quality and pharmacological potential of *R. astragali*.

Calycosin, calycosin-7-glucoside, formononetin, ononin, 7,2′-dihydroxy-3′,4′-dimethoxyisoflavan, and 3-hydroxy-9,10-dimethoxypterocarpan are classified as flavonoid compounds. Metabolomic pathway analysis revealed that phenylalanine participates in the phenylpropanoid biosynthesis pathway, leading to the formation of p-coumaroyl-CoA, which is the central precursor of flavonoid biosynthesis (Figure 6B). P-coumaroyl-CoA is subsequently involved in forming isoliquiritigenin, which serves as an intermediate for the synthesis of isoflavones. In parallel, p-coumaroyl-CoA can also give rise to other flavonoid subclasses, including flavonols and anthocyanins, through a series of enzymatic reactions. These results indicate that OMNBs treatment activates the phenylpropanoid and downstream flavonoid biosynthetic pathways, thereby promoting the accumulation and maintaining of bioactive functional flavonoids in *R. astragali* during postharvest storage.

## 4. Discussion

In this study, the effects of different concentrations of OMNBs treatments on blue mold control and quality preservation of fresh *R. astragali* inoculated with *P. polonicum* were firstly evaluated. The results indicate that 3 mg L^−1^ OMNBs displayed the best treatment influence. Furthermore, ozone treatment at concentrations either below or above 3 mg·L^−1^ showed no significant inhibitory effect on the growth of *P. polonicum* on the surface of *R. astragalus*. This may be related to the nonlinear dose-response effect of ozone in this system. At concentrations below 3 mg·L^−1^, the ozone dose may be insufficient to adequately induce stress responses and metabolic pathways in the host plant [36,37], resulting in no significant difference in efficacy compared to the control. Conversely, at concentrations above 3 mg·L^−1^, excessive ozone can cause notable oxidative damage, including membrane lipid peroxidation and protein denaturation [37,38], which compromises tissue integrity and leads to browning and quality deterioration in Astragalus. Therefore, an ozone concentration of 3 mg·L^−1^ demonstrated a relatively balanced antifungal efficacy under the present experimental conditions. Subsequently, the effects of the different treatments of O_3_, MNBs, and OMNBs on the physiological, pathological, and quality parameters of fresh *R. astragali* during storage were compared, and the influence of these treatments on the accumulation and modulation of functional components was conducted by metabolomics analysis, and then further verified by HPLC-MS. The results demonstrated that 3 mg L^−1^ OMNBs exhibited the most comprehensive preservation effect, not only effectively maintaining the physiological activity, quality attributes, and bioactive compounds of *R. astragali* but also effectively controlling the disease development and enhanced antioxidant capacity, thereby providing a promising, green, and efficient postharvest preservation strategy for *R. astragali*.

In terms of physiological quality, OMNBs treatment resulted in the lowest weight loss rate (Figure 3A) and the smallest decrease in firmness (Figure 3D). This study indicates that weight loss and firmness decline in *R. astragalus* are closely associated with respiratory intensity and surface moisture evaporation [39]. The significant inhibition of these indices by the OMNBs treatment observed in the present study suggests that this treatment may more effectively preserve tissue structure and internal water balance, thereby attenuating transpiration and respiratory metabolism, ultimately resulting in a better retention of weight and firmness. Respiration rate is a key indicator of metabolic activity driven by the decomposition of organic matter. Previous studies have shown that O_3_ treatment can lower respiratory intensity by modulating the activity of key enzymes in the ascorbic acid–glutathione (ASA-GSH) cycle [40]. In this study, the respiration rate under OMNBs treatment remained at a lower and more stable level (Figure 3C), suggesting that OMNBs effectively suppressed microbial proliferation and reduced respiratory substrate consumption. The decrease in respiration rate observed after fungal infection may be attributed to nutrient depletion caused by pathogenic fungi metabolism [41] and to the secretion of mycotoxins and other pathogenic factors that disrupt cellular and tissue structures of *R. astragali* [42], thereby weakening its respiratory capacity.

Browning degree is an important traditional parameter for evaluating the quality and commercial grade of *R. astragali*. In this study, the browning index of the OMNBs-treated *R. astragali* remained at a relatively lower level [Figure 3B], whereas that of the O_3_ treatment was markedly higher. This may be due to the strong oxidative potential of ozone, which promotes phenolic compound oxidation and accelerates enzymatic browning reactions [43]. However, the nanoscale characteristics of micro–nano bubbles substantially increase ozone solubility and stability in water [44], preventing localized oxidative damage caused by excessive ozone concentration. Furthermore, the encapsulated structure of MNBs allows for a slower release of ozone, mitigating oxidative intensity and reducing rapid cell destruction [45,46]. Consequently, OMNBs treatment effectively inhibited browning and preserved the visual and structural quality of *R. astragali* during storage.

ABTS and DPPH assays are widely used to evaluate the antioxidant capacity of medicinal and aromatic plants. In *R. astragali*, astragalus saponins, flavonoids, and anthocyanins represent the major non-enzymatic antioxidants [47,48], whose function is to scavenge free radicals generated during senescence and stress reactions [49]. In the present study, the decrease in ABTS and DPPH radical-scavenging activities during storage was primarily attributed to the degradation and transformation of antioxidant compounds in *R. astragali*. OMNBs treatment markedly suppressed the decline in both ABTS and DPPH scavenging rates (Figure 3E,F), indicating that OMNBs effectively delayed the loss of antioxidant activity. This preservation effect is likely associated with the inhibition of the oxidative degradation of saponins and flavonoids, which was consistent with the UPLC-MS results for active-component maintaining in this study.

The incidence rate reflects the extent of disease occurrence, whereas the disease index quantitatively integrates both incidence and severity. OMNBs treatment significantly attenuated the incidence rate and disease index in *R. astragali* (Figure 4A,B), whose influence can be attributed to the strong oxidizing capacity of ozone, which penetrates fungal and bacterial cell walls, alters membrane permeability, and causes cytoplasmic leakage, ultimately leading to microbial lysis and death [50]. In addition, ozone damages key cellular components such as membrane proteins, unsaturated fatty acids, enzymes, peptidoglycan, and intracellular nucleic acids [51]. Although both ozone and MNBs treatments inhibited microbial growth to some extent, their combination (OMNBs) exhibited superior performance. The slow-release property of MNBs extended ozone retention time in water [52], allowing for the continuous inhibition of pathogenic regeneration and preventing secondary infection caused by the rapid concentration decay typical of gaseous ozone treatments [53]. Consequently, OMNBs treatment significantly delayed mold growth and disease development of *R. astragali* during storage (Figure 4C). The antimicrobial effect is achieved through the combined action of ozone’s oxidative sterilization and the sustained release mediated by MNBs, which together ensure long-lasting protection.

Astragalosides I-IV are classified as triterpenoid saponins [54]. Metabolomic analysis indicated that terpenoids in *R. astragali* are mainly synthesized through the mevalonate (MVA) and methylerythritol phosphate (MEP) pathways (Figure 6A). The key intermediates acetyl-CoA, pyruvate, and glycerol 3-phosphate are primarily derived from amino acid degradation and glycolysis. In this study, OMNBs treatment up-regulated the accumulation of DL-serine and DL-valine. Given that these two amino acids are established precursors for the biosynthesis of pyruvate and acetyl-CoA [55,56], we speculate that their accumulation may contribute to elevated levels of these key metabolites, thereby promoting the associated metabolic processes. In the present study, OMNBs treatment up-regulated the accumulation of DL-serine and DL-valine, thereby promoting the formation of acetyl-CoA and pyruvate. Within the glycolytic pathway, the levels of glycerol 3-phosphate, D-glucose 6-phosphate, and D-fructose 6-phosphate were up-regulated under OMNBs treatment, but they were down-regulated under O_3_ exposure. This may be attributed to oxidative damage caused by ozone, which attacks unsaturated fatty acids in cell membranes, triggers lipid peroxidation chain reactions, and consequently reduces glycerol 3-phosphate levels, thereby inhibiting the MEP pathway [57]. In the MVA pathway, OMNBs treatment markedly up-regulated key intermediates involved in terpenoid biosynthesis, including 3-hydroxy-3-methylglutaryl-CoA, acetyl-CoA, mevalonate, and isopentenyl pyrophosphate (IPP), facilitating the formation of terpenoids. In contrast, IPP was down-regulated under MNBs and O_3_ treatments, which may have restricted terpenoid synthesis. Astragaloside IV was significantly down-regulated under O_3_ treatment, possibly due to oxidative conversion to astragalosides I–III [58]. Additionally, astragalosides II and III were markedly decreased under both OMNBs and O_3_ treatments, likely owing to degradation or transformation into astragaloside I, explaining the elevated contents of astragalosides I and IV observed in the OMNBs group.

Regarding the flavonoid biosynthetic pathway, OMNBs treatment specifically up-regulated phenylalanine and p-coumaroyl-CoA levels, thereby enhancing the phenylpropanoid metabolic flux and promoting the biosynthesis of downstream flavonoids. Moreover, OMNBs markedly inhibited the degradation of isoliquiritigenin and increased the production of isoflavones such as ononin. Calycosin was further glycosylated to form calycosin-7-glucoside, which may account for its higher accumulation in OMNBs-treated samples compared with other treatments.

## 5. Conclusions

This study demonstrated that treatment with 3 mg L^−1^ ozone micro–nano bubbles (OMNBs) effectively alleviated blue mold disease and maintained the postharvest quality of fresh *R. Astragali* inoculated with *P. polonicum*. Compared with the control, MNBs, and O_3_ treatments, OMNBs treatment significantly reduced weight loss, firmness decline, and respiration rate, and delayed the increases in incidence rate and browning index, as well as preserved the antioxidant capacity and contents of the bioactive functional components. Metabolomic analysis further indicated that OMNBs treatment was associated with enhanced biosynthesis of terpenoids and flavonoids, with corresponding upregulation observed in key metabolic pathways, including the mevalonate (MVA), methylerythritol phosphate (MEP), and phenylpropanoid pathways. These metabolic alterations were consistent with UPLC-MS results, confirming that OMNBs application significantly increased the accumulation levels of active compounds such as astragaloside I, astragaloside IV, ononin, and calycosin-7-glucoside.

Although this study confirms that OMNBs effectively inhibit postharvest blue mold development and preserve the bioactive composition of *R. astragali*, the molecular mechanisms underlying their regulation of terpenoid and flavonoid biosynthesis require further elucidation. In addition, the investigation employed a single treatment concentration without systematically examining the synergistic effects of ozone concentration, micro–nano bubbles parameters, and treatment duration. The impact of this treatment on the *R. astragalus*–pathogen interaction interface and microbial community structure also remains unexplored. Future studies should integrate multi-omics and molecular experimental approaches to further unravel the metabolic regulatory network and optimize process parameters to facilitate the practical application of this technology.

## Figures and Tables

**Table 1:** Quantitative results of metabolites.

Name	Adduct	Groups				
		Control	Treatment 1	Treatment 2	Treatment 3	Treatment 4
Astragaloside I	[M + H-H^2^O]+	1.85 × 10^7^	1.81 × 10^7^	2.29 × 10^7^	1.11 × 10^7^	2.47 × 10^7^
Astragaloside II	[M + Na]+	2.29 × 10^7^	1.02 × 10^7^	1.55 × 10^7^	9.71 × 10^6^	9.15 × 10^6^
Astragaloside III	[M + Na]+	2.26 × 10^7^	1.55 × 10^7^	1.72 × 10^7^	1.26 × 10^7^	1.10 × 10^7^
Astragaloside IV	[M + H]+	9.11 × 10^6^	1.74 × 10^7^	1.45 × 10^7^	1.59 × 10^7^	1.96 × 10^7^

## Data Availability

The original contributions presented in this study are included in the article. Further inquiries can be directed to the corresponding author.

## Appendix A

**Table A1:** Disease classification standard.

Disease Grading	Symptoms Described	The Degree of Pathogenicity
0	No symptoms	did not cause disease
1	The incidence area was only less than 10%, local slight browning, no mildew	low pathogenicity
3	The incidence area accounted for 11–30%, with a small amount of mildew and browning	low pathogenicity
5	The incidence area accounted for 31–50%, the mildew area became larger, and the browning color deepened	moderate pathogenicity
7	The incidence area accounted for 51–80%, mold accumulation present, and rot began	moderate pathogenicity
9	The incidence area accounted for 81–100%, the degree of decay was deepened, and pungent odor was produced	high pathogenicity

Note: for representative visual examples corresponding to each disease index category, please refer to Figure 2D or Figure 3A.

**Table A2:** Effects of different treatments on the content of active ingredients in *R. astragali*.

Group Storage Time					Active Ingredients Content (μg/g)						
		1	2	3	4	5	6	7	8	9	10
CK	7d	165.54 ± 6.09	73.39 ± 12.85	64.61 ± 14.63	13.66 ± 1.12	4.79 ± 0.72	47.49 ± 1.09	17.68 ± 0.34	0.69 ± 1.21	192.72 ± 12.52	2.35 ± 0.30
	14d	1278.49 ± 87.50	72.37 ± 12.85	53.34 ± 13.84	53.4 ± 13.86	12.34 ± 7.63	102.64 ± 39.23	59.95 ± 36.31	2.15 ± 0.45	216.26 ± 30.66	6.62 ± 4.01
	28d	804.73 ± 203.86	63.62 ± 26.93	53.94 ± 20.73	64.69 ± 14.65	17.14 ± 6.63	115.56 ± 9.87	67.8 ± 21.83	8.1 ± 0.83	384.3 ± 36.17	18.43 ± 1.73
	42d	667.01 ± 76.97	10.19 ± 2.80	14.92 ± 4.19	74.69 ± 14.65	22.87 ± 0.48	126.44 ± 32.81	38.62 ± 3.72	50.15 ± 16.83	489.85 ± 38.16	25.2 ± 9.70
	56d	537.59 ± 58.57	89.6 ± 21.63	110.94 ± 49.35	54.01 ± 20.76	14.37 ± 3.29	128.35 ± 19.26	34.64 ± 9.28	3.38 ± 0.88	119.77 ± 31.12	7.52 ± 2.06
MNBs	7d	321.64 ± 19.95	71.1 ± 10.02	111.35 ± 10.75	27.07 ± 4.06	4.08 ± 1.23	80.89 ± 7.16	31.77 ± 2.94	0.8 ± 0.24	36.02 ± 8.63	4.64 ± 0.39
	14d	621.2 ± 213.10	63.08 ± 12.86	24.87 ± 6.31	34.06 ± 3.20	10.28 ± 1.18	75.62 ± 18.02	38.51 ± 9.46	4.78 ± 0.43	282.92 ± 19.30	2.22 ± 0.54
	28d	957.46 ± 245.56	37.75 ± 5.92	24.69 ± 5.26	35.95 ± 6.53	14 ± 5.09	93.55 ± 5.80	79.37 ± 10.99	9.31 ± 0.43	346.66 ± 31.63	16.81 ± 1.07
	42d	1567.33 ± 159.98	20.08 ± 5.70	13.89 ± 5.55	111.49 ± 10.76	22.9 ± 2.16	100.71 ± 13.44	59.3 ± 20.45	36.28 ± 11.08	348.18 ± 28.83	28.54 ± 9.42
	56d	947.33 ± 51.88	61.05 ± 15.07	24.87 ± 6.31	62.96 ± 7.14	21.01 ± 2.97	114.46 ± 38.46	49.39 ± 6.77	6.44 ± 0.81	125.74 ± 15.82	12.22 ± 1.64
O_3_	7d	241.87 ± 31.56	74.13 ± 11.51	40.27 ± 3.49	13.9 ± 5.55	3.58 ± 0.46	67.01 ± 7.88	26.36 ± 3.27	0.78 ± 0.08	85.89 ± 13.06	0.96 ± 0.14
	14d	354.24 ± 105.83	47.57 ± 11.68	37.3 ± 1.98	21.99 ± 1.39	4.74 ± 0.59	75.99 ± 10.43	39.97 ± 2.35	1.23 ± 0.11	94.92 ± 12.67	1.41 ± 0.21
	28d	886.16 ± 90.77	43.11 ± 4.21	35.91 ± 6.52	24.89 ± 6.31	8.19 ± 0.08	97.52 ± 23.80	52.9 ± 7.44	13.53 ± 0.74	234.69 ± 8.72	25.33 ± 0.66
	42d	653.16 ± 115.42	23.32 ± 10.45	27.04 ± 4.06	39.89 ± 1.65	29.96 ± 0.68	108.35 ± 4.50	77.7 ± 12.15	25.39 ± 3.13	686.33 ± 107.38	42.63 ± 6.03
	56d	410.56 ± 26.72	63.08 ± 12.86	102.16 ± 36.25	44.52 ± 1.69	11.44 ± 2.99	175.85 ± 24.83	39.26 ± 9.45	3.23 ± 0.89	201.78 ± 49.21	4.86 ± 1.15
OMNBs	7d	715.63 ± 60.43	72.76 ± 4.76	74.24 ± 12.92	18.44 ± 6.90	3.63 ± 0.59	38.08 ± 5.05	11.27 ± 1.54	0.46 ± 0.09	120.71 ± 22.85	2.15 ± 0.31
	14d	1134.9 ± 198.02	63.63 ± 26.93	62.89 ± 7.40	23.18 ± 3.79	4.58 ± 0.82	68.25 ± 4.53	27.51 ± 5.49	1.16 ± 0.12	172.9 ± 23.03	2.49 ± 0.49
	28d	1589.15 ± 352.89	50.04 ± 5.73	36.1 ± 10.71	36.14 ± 10.73	12.21 ± 0.79	84.04 ± 18.47	96.52 ± 4.95	5.26 ± 1.12	257.62 ± 47.84	20.23 ± 3.88
	42d	1676.36 ± 721.85	11.99 ± 1.60	17.57 ± 3.79	128.63 ± 9.02	24.68 ± 3.30	114.55 ± 45.36	86.42 ± 10.58	23.57 ± 3.46	394.26 ± 29.08	57.45 ± 7.96
	56d	517.45 ± 66.62	44.7 ± 7.86	18.42 ± 6.89	136.18 ± 13.35	16.94 ± 0.63	191.3 ± 24.81	38.14 ± 2.58	3.34 ± 0.37	87.11 ± 5.62	4.2 ± 0.40

Note: 1: astragalus I, 2: astragalus II, 3: astragalus III, 4: astragaloside IV, 5: calycosin, 6: calycosin-7-glucoside, 7: ononin, 8: formononetin, 9: 7,2′-dihydroxy-3′,4′-dimethoxyisoflavan, 10: 3-hydroxy-9,10-dimethoxypterosanane. Data are presented as mean ± SD (*n* = 9).

**Table A3:** Percentage content of the top 20 metabolites.

Name	Group				
	Control	Treatment 1	Treatment 2	Treatment 3	Treatment 4
DL-arginine	10.29%	11.20%	8.99%	10.76%	10.90%
Choline cation	9.08%	8.36%	7.40%	7.14%	8.49%
Arginine	6.25%	5.14%	6.92%	5.01%	5.60%
Melibiose	7.24%	4.77%	5.26%	5.01%	5.49%
D-proline	3.79%	4.12%	3.60%	4.99%	3.96%
Canavanine	4.26%	3.32%	2.61%	4.79%	4.27%
Thr-Gly	2.49%	2.34%	2.97%	2.13%	2.27%
DL-malic acid	1.85%	2.67%	2.53%	2.40%	2.69%
D-turanose	2.75%	2.24%	2.27%	2.22%	2.13%
Oleamide	2.20%	2.38%	2.41%	2.22%	2.11%
2′-O-methyladenosine	3.45%	1.57%	1.75%	1.72%	1.78%
Palmitic acid	1.37%	1.50%	1.83%	1.60%	1.31%
D-(+)-trehalose	1.64%	1.21%	1.27%	1.21%	1.11%
Sucrose	1.10%	1.08%	1.04%	0.90%	0.94%
p-Aminobenzoic acid	0.98%	1.10%	0.95%	0.94%	1.10%
gamma-Aminobutyric acid	0.84%	0.83%	1.06%	1.00%	1.00%
4-Fluoropiperidine	0.89%	0.78%	1.27%	1.09%	0.46%
1,3,7-Trimethyluric acid	0.57%	0.97%	0.89%	0.86%	1.11%
Linoleic acid	0.59%	0.91%	0.85%	0.97%	0.93%
N-acetylglucosaminylasparagin	0.61%	1.03%	1.02%	0.36%	1.21%
Other	37.76%	42.49%	43.13%	42.69%	40.84%
